# Cytoreductive surgery and hyperthermic intraperitoneal chemotherapy in seven patients with peritoneal sarcomatosis from uterine sarcoma

**DOI:** 10.1002/ccr3.1491

**Published:** 2018-05-02

**Authors:** Armando Sardi, Carlos A. Muñoz‐Zuluaga, Michelle Sittig, Teresa Diaz‐Montes

**Affiliations:** ^1^ The Institute for Cancer Care Mercy Medical Center 227 St. Paul Place Baltimore 21202‐2001 Maryland

**Keywords:** Cytoreductive surgery, hyperthermic intraperitoneal chemotherapy, peritoneal sarcomatosis, uterine sarcoma

## Abstract

Peritoneal sarcomatosis from uterine sarcoma is a rare disease with no effective treatment and poor prognosis. Cytoreductive surgery with hyperthermic intraperitoneal chemotherapy (CRS/HIPEC) has successful results in peritoneal carcinomatosis from gastrointestinal/gynecological origins. We show that CRS/HIPEC is safe, feasible, and may benefit selected patients with peritoneal sarcomatosis from uterine sarcoma.

## Introduction

Uterine sarcoma (US) describes a group of rare malignant tumors arising from the uterine musculature or the connective tissue 1. US represents approximately 1% of all female genital tract malignancies and 3–9% of uterine cancers 2, 3. In 2016, 60,050 new cases of uterine cancer were estimated in the United States; among those, only 1600 cases were expected to be US 4.

The World Health Organization (WHO) classified the US histological subtypes into mesenchymal tumors (e.g., leiomyosarcoma, low/high‐grade endometrial stromal sarcoma, and undifferentiated uterine sarcoma) and mixed epithelial and mesenchymal tumors (adenosarcoma) 5. Eighty percent of US are represented by leiomyosarcoma and stromal sarcoma (51% and 30%, respectively), with the first histological subtype having the worst 5‐year survival (15–42% and 50–90%, respectively) 2, 3, 6.

Peritoneal sarcomatosis (PS) can be an aggressive primary presentation of US 7. It results from the intra‐abdominal spread of malignant disease involving the peritoneal surface. PS is characterized by an increased risk of recurrence and poor long‐term survival (median overall survival 6–15 months), indicating lack of an effective treatment 8, 9, 10. Classic therapeutic modalities such as radiotherapy, chemotherapy, and palliative surgery have not been shown to improve patient outcomes 7, 11, 12.

Cytoreductive surgery (CRS) with hyperthermic intraperitoneal chemotherapy (HIPEC) has been shown to be a promising treatment for the locoregional management of selected patients with US presenting with peritoneal dissemination 11, 13, 14, 15, 16. In the past, CRS/HIPEC has been successfully used in patients with peritoneal mesothelioma 17 and peritoneal carcinomatosis from both gastrointestinal 18, 19, 20, 21, 22 and gynecological 23, 24, 25, 26 origins; however, its use in US is still controversial 27, 28. We examined our experience with patients presenting with PS from US treated with CRS/HIPEC.

## Patients and Methods

A retrospective review of a prospective database of 647 patients who underwent CRS/HIPEC was carried out. Seven patients with PS arising from the uterus treated with CRS/HIPEC between May 2001 and November 2014 were identified.

All patients fulfilled the following criteria: histopathological diagnosis of PS from US, a feasible complete cytoreduction based on patient's history, physical examination, and computed tomography (CT) scans of the chest, abdomen, and pelvis, no evidence of extra‐abdominal disease, and a physical status score ≤III, according to the American Society of Anesthesiologists (ASA) classification 29. In all cases, histopathology reports from previous biopsies or surgeries were reviewed and diagnosis confirmed by CRS/HIPEC histopathology, at our institution.

Cytoreductive surgery/hyperthermic intraperitoneal chemotherapy techniques and perioperative care were carried out as previously described 30. The extent of disease was scored using the peritoneal cancer index (PCI) 31, and the volume of residual disease was graded according to the completeness of cytoreduction (CC) score 31. The chemotherapeutic agents used during HIPEC were melphalan (50 mg/m^2^) in cases with recurrent disease and doxorubicin (7 mg/m^2^) plus cisplatin (50 mg/m^2^) for primary malignancy. Postoperative complications were defined according to Dindo's Classification of Surgical Complications 32. The use of perioperative and postoperative chemotherapy or radiation therapy was recorded.

Follow‐up was carried out at 3 weeks, 3 months, and every 6 months thereafter, with CT scan of the chest, abdomen, and pelvis performed 1 month postoperatively, at 6‐month intervals for 5 years and yearly thereafter. Institutional Review Board approval was obtained for this retrospective review and a waiver of informed consent was granted.

Overall survival (OS) and progression‐free survival (PFS) were analyzed using the Kaplan–Meier method. OS and PFS were calculated from the time of CRS/HIPEC to the time of death and from the time of CRS/HIPEC to the time of recurrence, respectively. Patients without any events following CRS/HIPEC were censored at the time of the last follow‐up visit. Clinical data were described.

## Results

The median age at the time of diagnosis was 39 years with a range of 26–68 years. Two patients presented with class III obesity (body mass index [BMI] ≥40) and three patients were considered overweight (BMI ≥25) at the time of CRS/HIPEC. Five patients presented with ASA scores of II and two patients had ASA scores of III. Histopathological types were leiomyosarcoma (*n* = 4), adenosarcoma (*n* = 2), and endometrial stromal sarcoma (*n* = 1). Table 1 shows specific clinicopathologic characteristic of these cases**.**

**Table 1:** Patient characteristics prior to CRS/HIPEC

Patient	Age at Diagnosis (year)	BMI	ASA Class	Prior Surgeries	Chemotherapy and/or Radiotherapy prior to CRS/HIPEC	Primary/ Recurrent Uterine Sarcoma	FIGO Stage	Histopathological Subtype (Grade)	Time from initial surgery to first recurrence (months)	Time from initial surgery to CRS/HIPEC (months)
1	39	52.6	II	NA	NA	Primary	IVA	Leiomyosarcoma (HG)	NA	NA
2	49	21.3	III	TAH/BSO Debulking	Gemcitabine/docetaxel	Recurrent	IVA	Leiomyosarcoma (HG)	7	9
3	27	24.8	II	TAH/BSO Resection of pelvis mass	Brachytherapy	Recurrent	IIA	Endometrial stromal sarcoma (LG)	17	19
4	58	26.2	II	Lap: Hysterectomy/ Cholecystectomy Resection of pelvis mass	Gemcitabine/docetaxel Doxorubicin Trabectedin/pazopanib	Recurrent	IIIB	Leiomyosarcoma (HG)	26	47
5	68	26.7	II	TAH/BSO Debulking Debulking	Doxorubicin Gemcitabine/docetaxel Ifosfamide Pazopanib	Recurrent	IA	Adenosarcoma with sarcomatous overgrowth (LG)	11	39
6	26	25.6	II	Hysterectomy/ Omentectomy/ Pelvic lymphadenectomy	Gemcitabine/docetaxel	Recurrent	IIB	Leiomyosarcoma (HG)	46	46
7	38	50.0	III	TAH/BSO	Carboplatin/paclitaxel Ifosfamide	Recurrent	IIIB	Adenosarcoma with sarcomatous overgrowth (HG)	14	14

ASA, American Society of Anesthesiologists; BMI, body mass index; BSO, bilateral salpingo‐oophorectomy; CRS/HIPEC, cytoreductive surgery and hyperthermic intraperitoneal chemotherapy; FIGO, International Federation of Gynecology and Obstetrics; HG, high grade; Lap, laparoscopy; LG, low grade; NA, not applicable; TAH, total abdominal hysterectomy.

**Figure 1:**
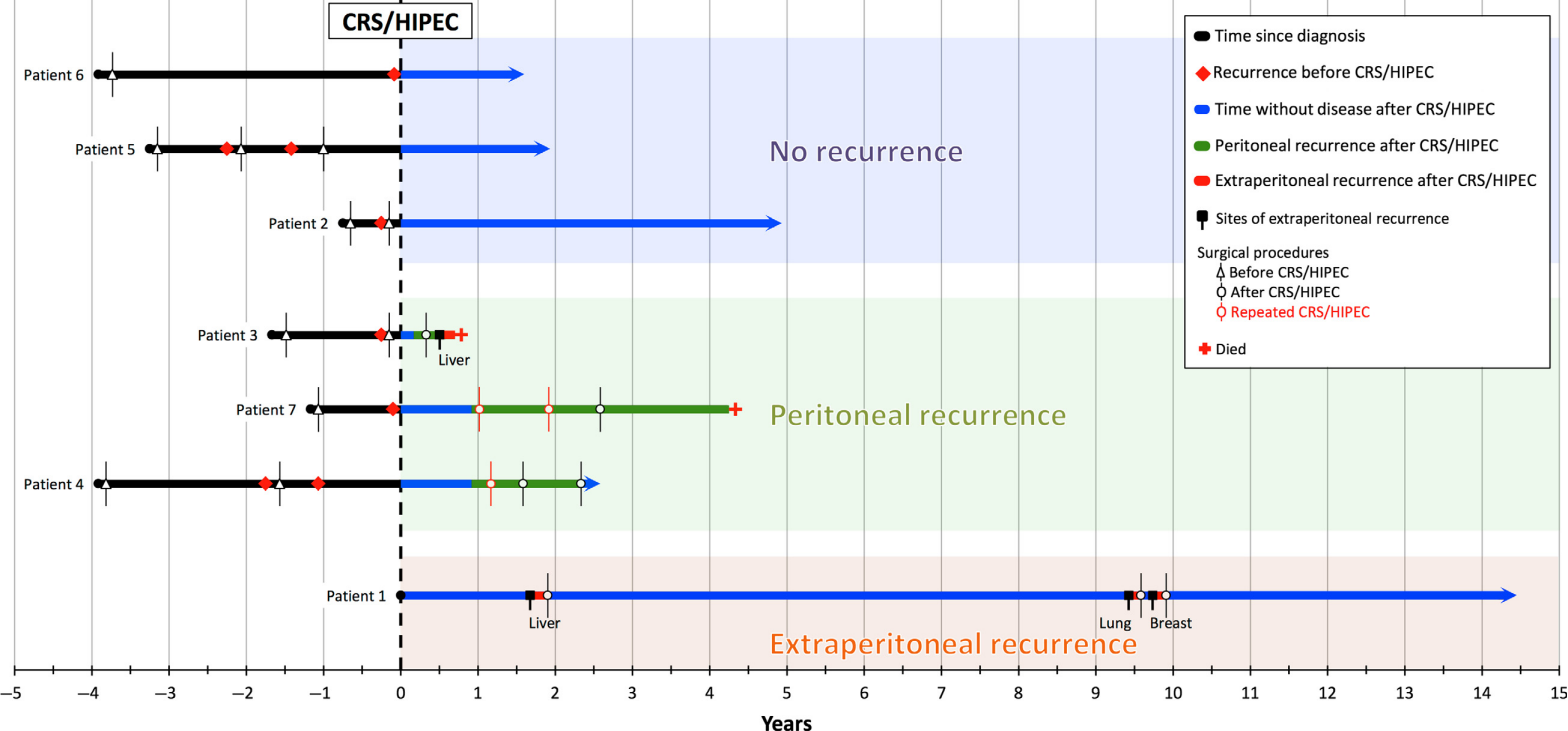
Patient timeline from initial diagnosis to last follow‐up/death. Summary of patient interventions categorized by recurrence type. CRS/HIPEC*,* cytoreductive surgery and hyperthermic intraperitoneal chemotherapy.

At the time of CRS/HIPEC, six patients had recurrent disease after treatment and one had primary disease (Fig. 1). All patients with recurrent US had at least one previous surgery and five patients received multiple chemotherapeutic agents prior to CRS/HIPEC. One patient received brachytherapy. Prior surgeries were performed in recognized centers for cancer care. FIGO stages were IA (*n* = 1), IIA (*n* = 1), IIB (*n* = 1), IIIB (*n* = 2), and IVA (*n* = 2), with leiomyosarcoma as the histopathological subtype presenting with the highest stage. Median time from initial surgery to first recurrence was 16 months (range 7–46 months) and from initial surgery to CRS/HIPEC was 29 months (range 9–47 months) (Table 1 and Fig. 1).

All patients underwent multiple resections during CRS/HIPEC in order to achieve a complete cytoreduction (Table 2). The mean PCI was 18 (range 7–29), and complete cytoreduction (CC‐0) was achieved in all cases. The mean length of CRS/HIPEC was 8 h (range 6–9 h), and the median length of hospital stay was 8 days (range 5–21 days).

**Table 2:** CRS/HIPEC characteristics and postoperative outcomes

Patient	Resections performed at CRS/HIPEC	PCI	CC Score	Intraperitoneal Chemotherapy	Length of surgery (hours)	Complications (Grade)	LOS (days)	Adjuvant therapy after CRS/HIPEC	Time to recurrence after CRS/HIPEC (months)	Site of recurrence	Status/Follow‐up since CRS/HIPEC (months)
1	TAH, L/R‐SO, TO, UTR, SSBR, L/R‐PP, RMSPI	16	0	Doxorubicin/cisplatin	6	No	5	Letrozole	20	Liver	NED/(172)
2	AWR, ITR, PTR, PCy, PV, L/R‐U, R‐ID, TO, L/R‐PP, Choly, RMSPI	22	0	Melphalan	8	Anemia Leukopenia (II)	15	NA	NA	NA	NED/(59)
3	AWR, L/R‐PP, TO, ICR, PTR, PCy, RMSPI, R‐U, R‐ID. Resection of tumor over rectosigmoid. Resection of internal iliac vessels (artery and vein)	25	0	Melphalan	8	Anemia Ileus (II)	7	NA	3	Pelvis	DOD/(8)
4	L/R‐PP, PC, APP, PTR, TO, PC, L‐SO, AWR, PLCR, PRPM, R‐ID, RMSPI. Resection of distal right ureter, partial resection of proximal stump of the right iliac vein	18	0	Melphalan	9	Leukopenia (II)	9	Anastrozole	11	Retroperitoneal, anterior abdominal wall	NED/(29)
5	UTR, AWR, AL (during 3 h), L/R‐PP, TO, Ce, SR, SSBR, PP, RMSPI. Excision of the tumor of left diaphragmatic peritoneum, resection of bilateral ovarian veins.	29	0	Melphalan	9	Pancytopenia Urinary tract infection (II)	21	NA	NA	NA	NED/(23)
6	PTR, L‐Col, L‐SO, L‐U, L‐ID, PLSR, TO	11	0	Melphalan	7	Intestinal obstruction^a^ (II)	8	Pelvic radiation	NA	NA	NED/(18)
7	AWR, TO, SR, PTR, RMSPI.	7	0	Melphalan	7	No	8	Tamoxifen Pelvic radiation^b^ Pazopanib	12	Pelvis	DOD/(53)

a Patient readmitted after discharge for intestinal obstruction, resolved with conservative management.

b Pelvic radiation as a treatment.

AL, adhesiolysis; APP, appendectomy; AWD, alive with disease; AWR, abdominal wall resection; CC, completeness cytoreduction; Ce, cecectomy; Choly, cholecystectomy; DOD, dead of disease; ICR, ileocolonic resection; ITR, intra‐abdominal tumor resection; L/R‐Col, left/right colectomy; L/R‐ID, left/right iliac dissection; L/R‐PP, left/right parietal peritonectomy; L/R‐SO, left/right salpingo‐oophorectomy; L/R‐U, left/right ureterolysis; LOS, length of stay; CRS/HIPEC, cytoreductive surgery and hyperthermic intraperitoneal chemotherapy; NA, no applicable; NED, not evidence of disease; PCe, partial cecectomy; PCI, peritoneal cancer index; PCy, partial cystectomy; PLCR, partial liver capsule resection; PLSR, partial liver segment resection; PP, pelvic peritonectomy; PRPM, partial resection of psoas muscle; PTR, pelvic tumor resection; PV, partial vaginectomy; RMSPI, removal of multiple small peritoneal implants; SR, sigmoid resection; SSBR, segmental small bowel resection; TAH, total abdominal hysterectomy; TO, total omentectomy; UTR, umbilical tumor resection.

There were five grade II postoperative complications, which included anemia/leukopenia/pancytopenia (*n* = 3), intestinal obstruction (*n* = 1), and urinary tract infection (*n* = 1) requiring transfusions/granulocyte colony‐stimulating factor, pharmacological treatment/total parenteral nutrition, and antibiotic therapy, respectively. There were no associated episodes of febrile neutropenia or sepsis. There was no hospital mortality (grade V complications) (Table 2).

**Figure 2:**
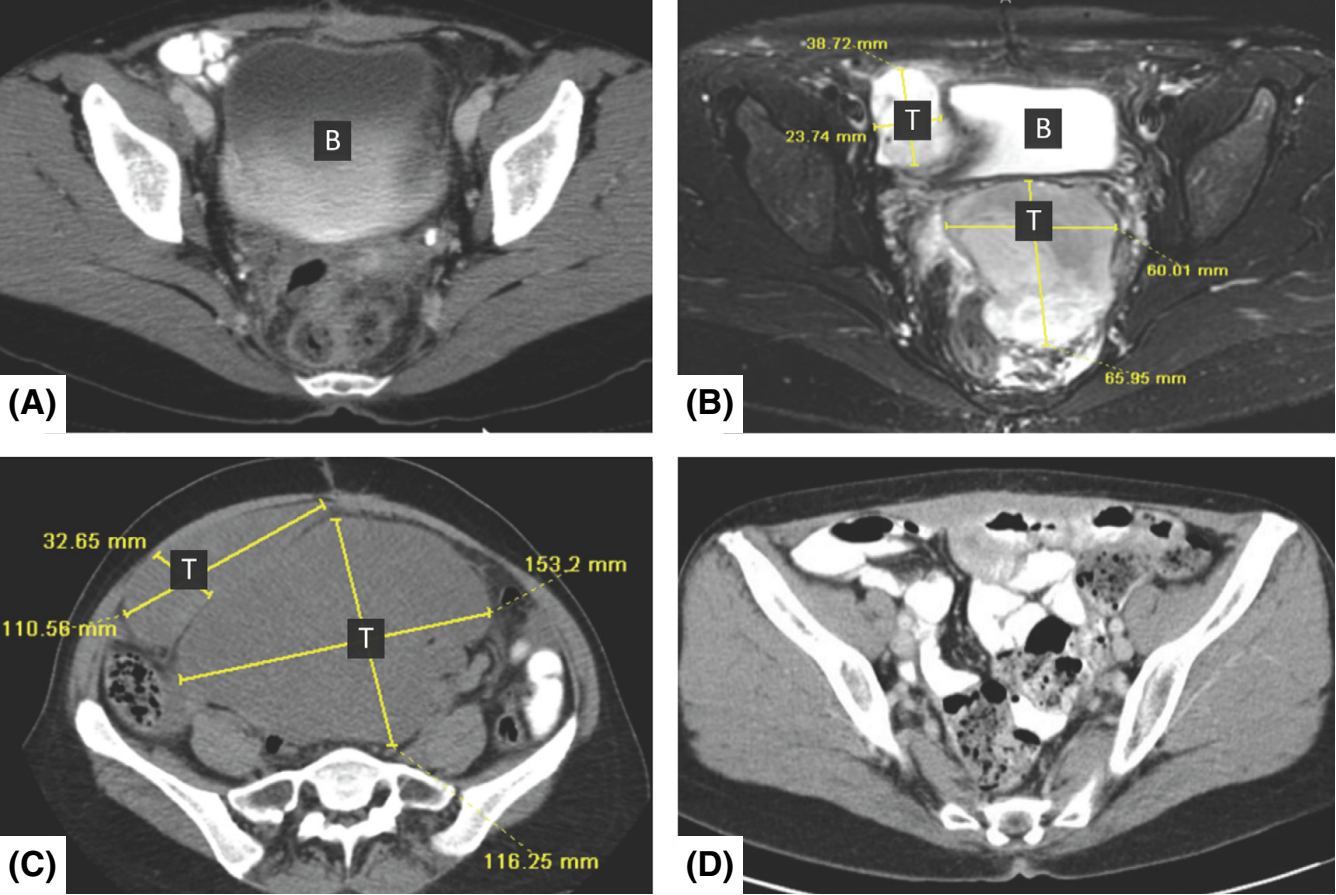
Rapid tumor recurrence. (A) Computed tomography (CT) scan of the pelvis 1 month after dubulking of pelvic recurrence and 8 months following TAH/BSO. No evidence of disease. (B) Magnetic resonance imaging (MRI) of the pelvis 1 month after image A showing two pelvic masses (65 × 60 mm and 23 × 38 mm). (C) CT scan of the pelvis 1 day before CRS/HIPEC showing rapidly growing tumors (116 × 153 mm and 110 × 32 mm) 4 weeks after MRI presented in image B. (D) CT scan of the pelvis, 5 years after CRS/HIPEC showing no evidence of disease recurrence. B, bladder; T, tumor; CRS/HIPEC, cytoreductive surgery and hyperthermic intraperitoneal chemotherapy.

Four patients (57%) recurred after CRS/HIPEC with a mean time of recurrence of 12 months (range 3–20 months) (Figs 1 and 2). The sites of recurrence included the pelvis (*n* = 2), the retroperitoneum and abdominal wall (*n* = 1), and the liver (*n* = 1).

Patient 1 initially recurred in the liver, followed by the lung and breast, all of which were successfully resected, demonstrating long‐term survival at 172 months after CRS/HIPEC and 54 months since the last resection of metastases. Patient 1 is currently alive with no evidence of disease and has never had peritoneal recurrence (Fig. 1).

Patient 3 relapsed 3 months after CRS/HIPEC with a small lesion (1.5 cm) in the anterior pelvis, which was managed by observation. She then presented 1 month later with small bowel obstruction requiring surgery. She presented 3 months afterward with new metastases to the liver, pelvis, abdomen, and subcutaneous soft tissue in the abdominal wall, subsequently dying 3 months after presentation of metastatic disease.

Patient 4 initially presented with enlarged uterine fibroids and underwent laparoscopic hysterectomy with morcellation in May 2010. Pathology reports were initially benign and follow‐up with physical examination every 6 months was unremarkable. Twenty‐six months later, she presented with unintentional weight loss and pelvic pressure with a palpable mass in the lower abdomen. CT scan confirmed a 20‐cm pelvic mass which was resected in July 2012. This surgery was complicated by an injury to the right ureter and the right iliac vein and artery, requiring an iliofemoral bypass graft and the re‐implantation of the right ureter in the bladder. Final pathology revealed leiomyosarcoma and review of the initial pathology was atypical smooth muscle neoplasm. The patient received 12 cycles of gemcitabine/docetaxel. She recurred 8 months later and an outside institution determined that surgical management would be of little benefit. As a result, she received doxorubicin/trabectedin/pazopanib as a second‐line therapy.

The patient sought a second opinion at our institution and was considered a candidate for CRS/HIPEC, which was performed in April 2014. Melphalan was the chemotherapeutic agent used during HIPEC and a complete cytoreduction (CC‐0) was achieved with PCI 18/0. Pathology was positive for estrogen receptors. Eleven months later, the patient recurred in the retroperitoneum and abdominal wall, and a second CRS/HIPEC with melphalan was performed in June 2015 (PCI 6/0 and CC‐0 were achieved). Four months after the second CRS/HIPEC, a follow‐up CT revealed a nodule localized lateral to the right psoas muscle in an area of initial surgery (resection of ureter and kidney mobilization). On December 2015, exploratory laparotomy was performed with excision of the nodule without complications. No peritoneal recurrence was seen. Six months later recurrence was again detected in the abdominal wall at an initial port site and resection of two lesions was performed. At the time of resection, no peritoneal disease was detected (September 2016). The patient is alive at 76 months since initial surgery, and 29 months since the first CRS/HIPEC.

Patient 7 had an adenosarcoma with sarcomatous overgrowth, which implies high‐grade malignancy 3, 5. Molecular testing showed aneuploid tumor with S‐phase fraction of 12.8% and a DNA index of 1.9 that suggested a shorter disease‐free survival 33, 34. The first CRS/HIPEC was performed for recurrence in the anterior pelvis, 14 months after TAH/BSO, with CC‐0. Twelve months later, the patient recurred with a 2‐cm right lower quadrant lesion close to the cecum, and a second CRS/HIPEC was performed with a PCI of 3/0 and CC‐0. The third recurrence appeared 11 months afterward with a mass at the level of the left pelvic brim. The patient underwent a third CRS/HIPEC with a PCI of 3/0 and CC‐0. New pathology reported positive estrogen and progesterone receptors. Chemosensitivity assay (ChemoFx assay, Precision Therapeutics Inc.) was nonresponsive to all regimens tested. The last recurrence was detected 8 months later with two lesions in the pelvis. She underwent a fourth CRS with incomplete cytoreduction and aborted HIPEC, due to unresectable encasement of the iliac arteries and the invasion of the inferior vena cava. Melphalan at 50 mg/m^2^ was the selected regimen in each HIPEC. The patient received radiotherapy (3000 cGy) and adjuvant therapy with pazopanib. The follow‐up CT scan revealed tumor at the right common iliac region involving the common iliac vein and right ureter. The patient developed severe anemia, hydronephrosis/renal failure, and died 53 months after the initial CRS/HIPEC.

Patients 2, 5, and 6 have never had recurrence with follow‐up of 59, 23, and 18 months, respectively (Fig. 1).

**Figure 3:**
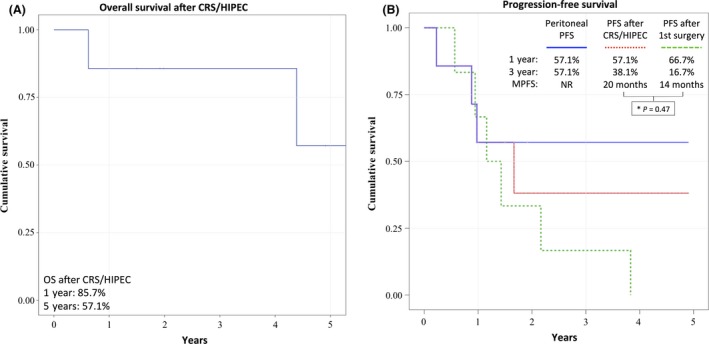
Kaplan–Meier curves showing (A) overall survival and (B) progression‐free survival of patients after CRS/HIPEC. CRS/HIPEC, cytoreductive surgery and hyperthermic intraperitoneal chemotherapy; OS, Overall survival; PFS, progression‐free survival; MPFS, median progression‐free survival; NR, not reached. *The differences were not significant between the groups. PFS after first surgery and after CRS/HIPEC with *P* = 0.47.

There are five patients alive without evidence of disease at 172, 59, 29, 23, and 18 month follow‐up (Table 2 and Fig. 1). The OS rate at 1 and 5‐years was 86% and 57%, respectively (Fig. 3A). The PFS at 1 and 3 years after the initial surgery was 67% and 17%, respectively, with median PFS (MPFS) of 14 months. The PFS at 1 and 3 years after CRS/HIPEC was 57% and 38%, respectively, with MPFS of 20 months. There was not a statistically significant difference between the PFS after first surgery in comparison with PFS after CRS/HIPEC (*P* = 0.47). Peritoneal PFS at 3‐years was 57% (Fig. 3B).

## Discussion

Uterine sarcoma is a rare and aggressive disease, representing 3–9% of uterine cancers 2, 3, 35. This malignancy has poor prognosis due to its biological behavior, high rates of recurrence, and ineffective treatment with the current therapeutic modalities 8, 9, 10, 35. Progression to PS frequently occurs after initial treatment 4, 11, 36; however, PS may be present at the first presentation of US 9, 11. There is no standard of care treatment for PS 9, 37. Chemotherapy (e.g., gemcitabine/docetaxel, or doxorubicin/ifosfamide) with or without radiation has low response rates (25–50%) 38, 39, 40 and surgical intervention is often only palliative 37. Surgery is required to manage symptoms such as intestinal obstruction, gastrointestinal bleeding, and perforation with short‐term benefits 14.

Cytoreductive surgery with HIPEC has been proposed as an optional therapy for PS based on the successful results of CRS/HIPEC in peritoneal carcinomatosis from appendiceal 18, 19, 20, 30, 41, 42, colorectal 21, 43, gastric 22, recurrent ovarian 23, 24, 26, and endometrial 25, 44 cancer origins, as well as peritoneal mesothelioma 17. Berthet et al., reported the use of CRS/HIPEC in 30 patients with PS, of which four were US. The 5‐year survival was 39% compared to 0% in patients who only received cytoreductive surgery. Mortality in the CRS/HIPEC group was 3% compared to 15% in the cytoreductive surgery alone group 14.

Rossi et al., performed CRS/HIPEC in 60 patients with PS, of which 12 had PS arising from US. In all patients, they estimated a median OS of 36 months and a median time to local disease recurrence of 24 months. Morbidity was 33% without postoperative deaths 27. Baratti et al. conducted a study with 37 patients diagnosed with PS and treated with CRS/HIPEC. Eleven patients with PS from US demonstrated a longer survival (median OS of 29.5 months) and higher local–regional progression‐free survival (median of 15 months) compared to the other patients with PS. Among all patients studied, the median OS was 26.2 months and the 5‐year OS was 24.3%. Morbidity was 21.6% and operative mortality 2.7% 28. Kusamura et al., reported the first homogeneous series of US treated by CRS/HIPEC. Ten patients with primary or recurrent disease underwent CRS/HIPEC with cisplatin/doxorubicin or cisplatin/mitomycin‐C, with no surgical complications, toxicity, or perioperative mortality. Reported 5‐year OS of 65% and 5‐year PFS of 30% were encouraging, suggesting that CRS/HIPEC might offer the best results in patients with PS from US 45. Prognostic factors after CRS/HIPEC include the degree of cytoreduction, histopathology/tumor grade, and tumor load, with complete cytoreduction as the most important factor positively affecting overall and progression‐free survival 11, 14, 27, 28, 46, 47, 48.

It is worth noting that laparoscopic resection of uterine tissue using power morcellators, especially in large and symptomatic uterine fibroids, in peri‐ or postmenopausal females as inpatient #4, may increase the risk of developing peritoneal dissemination of US 49, 50, 51. Sugarbaker et al., recorded six patients with disseminated uterine leiomyosarcoma after morcellation and demonstrated that early intervention with CRS/HIPEC plus early postoperative intraperitoneal chemotherapy (a multidrug regimen) was associated with a lesser extent of disease, less morbidity and without mortality 52.

Some authors have used a diagnostic algorithm based on patient age, LDH levels, endometrial cytological findings, and MRI to determine if morcellation is appropriate;^53^, ^54^, ^55^ however, others in a meta‐analysis have indicated that morcellation increases the overall and intra‐abdominal recurrence rate as well as death rate in uterine leiomyosarcomas 49. Bogani also found that very large masses and masses associated with uterine bleeding correlated with US in about 20% and 42%, respectively 50, 54. Therefore, comprehensive preoperative workup is desirable including detailed histopathology 55. If sarcomatosis is suspected, early referral for CRS/HIPEC may improve the prognosis and reduce morbidity 50, 52.

In our series, the 5‐year survival rate was 57% with a median progression‐free survival of 20 months. Seventy‐one percent (*n* = 5) of patients remain free of peritoneal disease, and interestingly, peritoneal recurrence was not seen even in the presence of distant metastases (Patient 1). Taking into consideration that the peritoneal cavity is the second most frequent site of metastasis (41%) after lung (74%), followed by bone (33%), and liver (27%) metastasis 56, this finding supports the use of CRS/HIPEC as a therapeutic locoregional approach which may significantly decrease the likelihood of developing recurrent intraperitoneal disease, and eventually may change the metastatic pattern of this malignancy.

We have found that different characteristics in the presentation and technical approach are needed for performing CRS/HIPEC in patients with PS from US when compared to other malignancies. Frequently, within the first few months following initial surgery, rapid tumor growth occurs and patients present with large symptomatic masses (Fig. 2). Secondly, these tumors appear to have less involvement in the upper abdomen and are less infiltrative into the bowel making extensive bowel resections less common. Thirdly, extensive peritonectomies are not usually needed in sarcomatosis, although extensive pelvic compromise is common requiring pelvic side wall, iliac vessel, and ureter resections, which are the most challenging components of the surgery. The majority of these patients have had prior extensive and often, multiple pelvic surgeries and wide field radiation, making resections difficult due to the degree of pelvic involvement. Finally, selecting the appropriate agents for intraperitoneal chemotherapy is a major consideration, especially in recurrent disease. Sarcomas for the most part are considered chemoresistant 57 and melphalan has been described as a successful agent in the treatment of aggressive chemoresistant neoplasms, such as soft tissue sarcomas and melanomas of the extremities when combined with hyperthermia 58, 59, 60, 61. Alkylating agents such as melphalan, cyclophosphamide, and ifosfamide had the highest activation energy by hyperthermia 62, 63, with melphalan having the greatest thermal enhancement cytotoxicity and drug penetration into tumor 64, 65.

Response to cytotoxic therapies in patients with advanced or recurrent uterine leiomyosarcoma has been limited with objective response rates from 27% to 53%, median PFS from 4.4 to 6.7 months, and median OS from 14.7 to 17.9 months, with combination gemcitabine plus docetaxel as the superior treatment approach 66, 67, 68, 69. Furthermore, novel targeted agents such as pazopanib, trabectedin, and olaratumab have shown objective response rates that ranged from 6% to 18.2% and PFS from 4.6 to 6.6 months, with an encouraging median OS of 26.5 months with olaratumab plus doxorubicin compared with 14.7 months with doxorubicin alone (*P* = 0.0003) 70, 71, 72. Traditionally, cisplatin and doxorubicin have been used in the treatment of PS in patients undergoing CRS/HIPEC; however, we have used melphalan for recurrent tumors of the gastrointestinal and gynecologic tracts with good results 73. At our institution, melphalan is also the primary drug of choice for primary peritoneal sarcomatosis, although no comparable studies are available.

Overall, CRS/HIPEC has been a well‐tolerated procedure in our patients with few complications. Grade II complications were treated with pharmacologic therapies and myelosuppression was the most common complication in 4 (40%) of 10 procedures. This is a known side effect of melphalan therapy 74 and was successfully managed in all patients with filgrastim treatment. There were no infectious complications or mortality associated with myelosuppression.

Following CRS/HIPEC, four patients recurred and currently five of the seven patients are alive without evidence of disease, with a 5‐year survival rate of 57%. This implies that HIPEC played an important role, although that role cannot be specifically defined at this time. Clearly, CRS is the most important component of the procedure and adherence to cytoreductive principles with complete surgical resection is the key in surgical management. According to the initial operative reports, all patients received a complete cytoreduction with no visible disease remaining. All procedures were performed at major cancer centers by experienced gynecologic oncologists; however, it should be kept in mind that the gynecologic oncology group (GOG) defines optimal cytoreduction as <10 mm 75, while surgical oncologist considers <2.5 mm as the optimal goal for completeness of cytoreduction 76. A dual surgical approach (surgical oncologist and gynecologic oncologist) may be beneficial for CRS/HIPEC in PS, as each surgeon can operate in their anatomic fields of specialization and work as a complementary team to achieve the best surgical results for the patient.

## Conclusion

Cytoreductive surgery/hyperthermic intraperitoneal chemotherapy can be safely performed in specialized centers and is a feasible treatment modality for PS from US, with complete cytoreduction, low morbidity, and promising survival, among carefully selected patients. We are currently conducting a worldwide review of cytoreductive surgery and hyperthermic intraperitoneal chemotherapy in peritoneal sarcomatosis from uterine sarcoma to better understand the role of HIPEC as a treatment for this disease.

## Authorship

AS: study concept and design, data interpretation, and critical manuscript revision. CAM‐Z: acquisition of data, analysis and interpretation of data, manuscript development, drafting, and critical manuscript revision. MS: acquisition of data, drafting, and critical manuscript revision. TD‐M: study concept and design, interpretation of the data, and critical manuscript revision.

## Conflict of Interest

The authors declare that there is no conflict of interest regarding the publication of this article.
